# Development of a Medical Social Media Ethics Scale and Assessment of #IRad, #CardioTwitter, and #MedTwitter Posts: Mixed Methods Study

**DOI:** 10.2196/47770

**Published:** 2024-03-27

**Authors:** Vongai Christine Mlambo, Eric Keller, Caroline Mussatto, Gloria Hwang

**Affiliations:** 1 School of Medicine Stanford University Palo Alto, CA United States; 2 Division of Interventional Radiology Stanford Health Care Stanford, CA United States; 3 School of Medicine University of Kansas Medical Center Kansas City, KS United States

**Keywords:** ethics, social media, conflict of interest, interventional radiology, X, Twitter, cardiology, privacy, ethical issues, medical social media, prevalence, professional, professionalism

## Abstract

**Background:**

Social media posts by clinicians are not bound by the same rules as peer-reviewed publications, raising ethical concerns that have not been extensively characterized or quantified.

**Objective:**

We aim to develop a scale to assess ethical issues on medical social media (SoMe) and use it to determine the prevalence of these issues among posts with 3 different hashtags: #MedTwitter, #IRad, and #CardioTwitter.

**Methods:**

A scale was developed based on previous descriptions of professionalism and validated via semistructured cognitive interviewing with a sample of 11 clinicians and trainees, interrater agreement, and correlation of 100 posts. The final scale assessed social media posts in 6 domains. This was used to analyze 1500 Twitter posts, 500 each from the 3 hashtags. Analysis of posts was limited to original Twitter posts in English made by health care professionals in North America. The prevalence of potential issues was determined using descriptive statistics and compared across hashtags using the Fisher exact and *χ*^2^ tests with Yates correction.

**Results:**

The final scale was considered reflective of potential ethical issues of SoMe by participants. There was good interrater agreement (Cohen κ=0.620, *P*<.01) and moderate to strong positive interrater correlation (=0.602, *P*<.001). The 6 scale domains showed minimal to no interrelation (Cronbach α=0.206). Ethical concerns across all hashtags had a prevalence of 1.5% or less except the conflict of interest concerns on #IRad, which had a prevalence of 3.6% (n=18). Compared to #MedTwitter, posts with specialty-specific hashtags had more patient privacy and conflict of interest concerns.

**Conclusions:**

The SoMe professionalism scale we developed reliably reflects potential ethical issues. Ethical issues on SoMe are rare but important and vary in prevalence across medical communities.

## Introduction

The digital footprint of clinicians on social media has increased over the past 10 years with an estimated 90% and 65% of clinicians using social media for personal and professional purposes, respectively [1]. Medical social media (SoMe) has blossomed, offering clinicians opportunities to collaborate across distances, debate treatment approaches for challenging cases, and engage in public health advocacy [2-4]. However, this rapid integration of social media in health care has outpaced guidance that counsels on how to avoid ethical concerns that can occur with SoMe [2].

The risks of SoMe have not gone unnoticed. Several professional organizations have released statements outlining guiding principles for online clinician behavior, including the American Medical Association and the Federation of State Medical Boards [5,6]. There have also been opinion pieces and recommendations published within various specialties such as neurology, dermatology, and vascular surgery [7-9]. Guidelines and opinion pieces are helpful starting points but may not address subtle but important breaches in professionalism [10] and may fail to resonate with the majority of users’ experiences and values [2].

A few studies have assessed the prevalence of issues such as violations of the HIPAA (Health Insurance Portability and Accountability Act) [10]. However, the potential issues are much broader than explicit patient privacy violations [10,11]. This study sought to develop a more complete scale of ethical issues related to medical SoMe to provide empirical data on these issues. The authors hypothesized that a scale could be developed that captures the most salient ethical issues with good interrater agreement and correlation. The authors also hypothesized that applying such a scale would find that the prevalence of issues was small and varied across different professional groups.

## Methods

### Scale Development

This study was approved by the Stanford University Institutional Review Board (eProtocol 60351). An initial draft of the scale was developed based on medical professionalism in the new millennium: a physician charter created by the American Board of Internal Medicine Foundation, American College of Physicians Foundation, and the European Federation of Internal Medicine as well as a study by Chandratilake et al [12] assessing definitions of medical professionalism across cultures [13]. These sources were selected to attempt to define medical SoMe ethics that would be reflective of common definitions of medical professionalism. The initial draft consisted of 5 criteria rated on a 3-point scale: no ethical concern (0), potential ethical concern (1), and clear ethical concern (2). The 3-point scale was selected to reflect a concept raised by both initial sources that ethical issues occur on a continuum, allowing the scale to also capture less overt violations of professionalism.

The initial scale was then vetted for validity via semistructured cognitive interviewing with a group of clinicians and trainees [14]. Interviewees were recruited via email and were primarily a convenience sample at the authors’ institutions. They were invited to provide feedback on a draft of the scale, which included fabricated posts and example scoring for demonstration. Purposeful recruiting was used to ensure that interviewees were diverse in terms of specialty, training level, and gender identity. Iterative adjustments were made to the initial scale based on interviewee feedback until additional interviews continued suggesting that the scale was reflective of interviewee perceptions of potential ethical issues related to medical SoMe. This occurred after 11 interviews with interviewees from 6 different specialties whose demographics are shown in Table 1.

**Table 1:** Demographic characteristics of interviewees (N=11).

Characteristic		Interviewees, n (%)
**Training level**		
	1st-year MD^a^	2 (18)
	2nd-year MD	0 (0)
	3rd-year MD	2 (18)
	4th+ year MD	1 (9)
	1st-year resident	0 (0)
	2nd-year resident	1 (9)
	3rd+ year resident	1 (9)
	Attending	4 (36)
**Institution**		
	Stanford University School of Medicine	8 (73)
	University of California San Diego	1 (9)
	University of Kansas Medical Center	2 (18)
**Specialty**		
	Anesthesiology	2 (18)
	DR^b^/IR^c^	1 (9)
	Emergency medicine	1 (9)
	Primary care	1 (9)
	Psychiatry	1 (9)
	Otolaryngology	1 (9)
	Undeclared	4 (36)
**Sex**		
	Female	7 (64)
	Male	4 (36)

^a^MD: Doctor of Medicine.

^b^DR: Diagnostic Radiology.

^c^IR: Interventional Radiology.

The vetted scale scored posts on 6 domains, using the same 3-point scale (Table 2). Scale item interrelation as well as scale interrater agreement and correlation were assessed by having 2 researchers use the scale to independently rate 50 random posts each from #MedTwitter between June 15, 2021, and August 15, 2021, with an overlap of 10 tweets. Posts were identified using the Healthcare Hashtag Project (Symplur, LLC). The interrelation of scale items was assessed via Cronbach α. Interrater agreement was assessed via Cohen κ and interrater correlation was assessed via Spearman correlation coefficient, assuming a nonlinear relationship. An α of <.05 was predefined as statistical significance.

**Table 2:** Medical social media professionalism scale.

Principle			Score				
			0=no concern		1=minor concern		2=major concern
**Patient privacy**							
	Does the post maintain patient privacy by applying appropriate safeguards for patient information and removing patient identifiers?	Post omits HIPAA^a^ identifiers and any other details that in combination would enable patient identification.		Post omits HIPAA identifiers but uses information that could potentially allow for patient identification, particularly when combined with the author’s known practice location, medical specialty, or rarity of medical condition.		Post uses one or more HIPAA identifiers that allows for easy identification.	
**Patient dignity**							
	Does the post treat patients with respect and avoid the use of degrading language or images?	Post treats patients as individuals worthy of respect and does not demean the patient in any way.		Post contains references, images, or language that could be negatively construed such that some may take offense.		Post is objectifying or dehumanizing, treating patients as being of lesser intelligence or caliber.	
**Information accuracy**							
	Is the information medically accurate with no counterfactual, exaggerated, or otherwise misleading content?	Information in the post is reasonably supported by current evidence and does not make superlative claims.		Information in the post is ambiguous or exaggerated in a manner that could lead to misinterpretation.		Information in the post is overtly sensational and makes baseless claims.	
**Conflict of interest**							
	Is the post unduly influenced by ulterior motives for private gain without proper acknowledgment or disclosure in a way that could affect information accuracy?	The post does not promote or endorse products or services without an appropriate declaration of any associated financial ties.		The post promotes or endorses products or services without a declaration of conflicts, however, it does not make authoritative claims about these products.		The post promotes or endorses products or services without a proper declaration of conflicts and also makes authoritative claims about these products.	
**Justice and equity**							
	Is the text or images in the post discriminatory based on race, gender, socioeconomic status, ethnicity, religion, sexual orientation, or any other social category and does the post promote further inequities in health care?	The post does not express or imply any discriminatory sentiments or propagate a stance that either sustains or widens inequities in health care.		The post contains ideas associated with stereotypes or broad generalizations *without* suggesting the differential treatment of individuals based on these stereotypes.		The post explicitly expresses sentiments that are discriminatory and is a proponent for the differential treatment of individuals based on these prejudiced notions.	
**Interprofessional respect**							
	Does the post treat colleagues and other health care professionals with respect and avoid the use of stereotypes, mockery, and incivility?	Post treats colleagues and other health care professionals with esteem and does not demean them in any way.		Post contains references, images, or language that could be negatively construed by other colleagues as offensive.		Post clearly mocks or disrespects colleagues, portraying them as inferior or of lesser intelligence or caliber.	

^a^HIPAA: Health Insurance Portability and Accountability Act.

### Evaluation of Posts

The validated scale was then used to assess the prevalence of ethical issues among posts using 3 distinct hashtags: #MedTwitter, #IRad, and #CardioTwitter. These were selected as they are the most frequently used hashtags among the general medical community, interventional radiologists, and cardiologists, respectively, as indicated by the number of posts per day for each hashtag on the Symplur software. Interventional Radiology (IR) and cardiology were selected to provide examples of more specialty-specific posts to contrast with #MedTwitter as they are primarily used by physician specialists in those fields to discuss more expert medical content compared to #MedTwitter. Posts were limited to those in English posted by individuals (rather than societies or bots) who are clinicians or health care trainees in North America between December 10, 2021, and January 10, 2022. Retweets were also excluded. A total of 1500 posts were analyzed, 500 from each hashtag. Data were analyzed using descriptive statistics as well as Fisher exact tests and *χ*^2^ tests with Yates correction to compare the prevalence of ethical issues across hashtags. These statistical tests were selected to adjust for the low rates of ethical issues. All statistical analyses were performed using SPSS software (IBM, Inc).

### Ethical Considerations

All procedures were approved by the Stanford University Institutional Review Board (IRB#: 60351) and were per the legal and ethical standards of the responsible committee on human experimentation institutionally. Additionally, we adhered to local, national, regional, and international laws and regulations regarding the protection of personal information, privacy, and human rights.

## Results

### Scale Development

Cognitive interviewing supported the validity of the initial 5 domains. However, the initial interviewees felt the initial scale did not address interspecialty and inter–health care professional cyberbullying, leading to the addition of interprofessional respect as a 6th domain. Interviewees also suggested the addition of language to better delineate a minor concern (1) rating from a major concern (2) rating. Subsequent interviews confirmed that the 6-domain scale, each rated from 0 to 2, was reflective of their perceptions of SoMe ethics.

The scale demonstrated good interrater agreement (Cohen κ=0.620, *P*<.01) and moderate to strong positive correlation between the scores given by the independent raters (Spearman correlation coefficient=0.602, 95% CI 0.515-0.677; *P*<.001). The scale domains showed minimal to no interrelation (Cronbach α=0.206).

### Evaluation of Posts

Application of the scale to 1500 Twitter posts showed that ethical concerns across all 6 domains were infrequent with the majority in the range of 0.2% (n=1) to 1.2% (n=6). Further, 1 exception was a minor conflict of interest concern among posts using #IRad, which demonstrated a prevalence of 3.6% (n=18). Relative to posts using #MedTwitter, posts using #IRad or #CardioTwitter were more likely to have patient privacy concerns (n=7, 1.4% vs 0%, *P*=.02; n=6, 1.2% vs 0%, *P*=.04; respectively). Posts using #IRad were also more likely to have conflicts of interest concerns relative to #MedTwitter and #CardioTwitter (n=18, 3.6% vs n=3, 0.6%, *P*<.001; n=18, 3.6% vs n=4, 0.8%, *P*=.005; respectively). Issues related to interprofessional respect were also more prevalent in #IRad posts than #CardioTwitter (n=8, 1.6% vs n=1, 0.2%, *P*=.04) but similar to #MedTwitter (n=8, 1.6% vs n=6, 1.2%, *P*=.79). As a result, across all domains, #IRad posts had the greatest overall prevalence of ethical concerns. Table 3 summarizes the prevalence of ethical concerns by hashtag and domain and Tables 4-6 summarize comparisons between hashtags.

**Table 3:** Prevalence of ethical concerns on medical social media by hashtag (N=500).

		No issue (0), n (%)	Minor concern (1), n (%)	Major concern (2), n (%)
**MedTwitter prevalence**				
	Patient privacy	500 (100)	0 (0)	0 (0)
	Patient dignity	495 (99)	3 (0.6)	2 (0.4)
	Information accuracy	497 (99.4)	2 (0.4)	1 (0.2)
	Conflict of interest	500 (100)	0 (0)	0 (0)
	Justice and equity	499 (99.8)	1 (0.2)	0 (0)
	Interprofessional respect	494 (98.8)	4 (0.8)	2 (0.4)
**IR^a^ prevalence**				
	Patient privacy	493 (98.6)	6 (1.2)	1 (0.2)
	Patient dignity	497 (99.4)	1 (0.2)	2 (0.4)
	Information accuracy	497 (99.4)	2 (0.4)	1 (0.2)
	Conflict of interest	482 (96.4)	18 (3.6)	0 (0)
	Justice and equity	500 (100)	0 (0)	0 (0)
	Interprofessional respect	492 (98.4)	7 (1.4)	1 (0.2)
**Cardiology prevalence**				
	Patient privacy	494 (98.8)	6 (1.2)	0 (0)
	Patient dignity	499 (99.8)	1 (0.2)	0 (0)
	Information accuracy	500 (100)	0 (0)	0 (0)
	Conflict of interest	496 (99.2)	2 (0.4)	2 (0.4)
	Justice and equity	500 (100)	0 (0)	0 (0)
	Interprofessional respect	499 (99.8)	1 (0.2)	0 (0)

^a^IR: Interventional Radiology.

**Table 4:** Comparison of ethical concerns on medical social media by hashtag^a^: #IRad vs #MedTwitter.”

		#IRad, n (%)	#MedTwitter, n (%)		Fisher exact *P* value		Chi-squared with Yates correction *P* value
**#IRad vs #MedTwitter**							
	Patient privacy	7 (1.4)^b^	0 (0)^b^	.02^b^		.02^b^	
	Patient dignity	3 (0.6)	5 (1)	.73		.72	
	Information accuracy	3 (0.6)	3 (0.6)	≥.99		≥.99	
	Conflict of interest	18 (3.6)^b^	0 (0)^b^	<.001^b^		<.001^b^	
	Justice and equity	0 (0)	1 (0.2)	≥.99		.32	
	Interprofessional respect	8 (1.6)	6 (1.2)	.79		.79	

^a^Comparisons reflect the composite of major and minor concerns for each scale criterion. *P*<.05 on a 2-tailed analysis was considered significant.

^b^Comparisons that are significant.

**Table 5:** Comparison of ethical concerns on medical social media by hashtag^a^: #CardioTwitter vs #MedTwitter.”

		#CardioTwitter, n (%)	#MedTwitter, n (%)	Fisher exact *P* value	Chi-squared with Yates correction *P* value
**#CardioTwitter vs MedTwitter**					
	Patient privacy	6 (1.2)^b^	0 (0)^b^	.03^b^	.04^b^
	Patient dignity	1 (0.2)	5 (1)	.22	.22
	Information accuracy	0 (0)	3 (0.6)	.37	.62
	Conflict of interest	4 (0.8)	0 (0)	.22	.37
	Justice and equity	0 (0)	1 (0.2)	≥.99	.32
	Interprofessional respect	1 (0.2)	6 (1.2)	.12	.13

^a^Comparisons reflect the composite of major and minor concerns for each scale criterion. *P*<.05 on a 2-tailed analysis was considered significant.

^b^Comparisons that are significant.

**Table 6:** Comparison of ethical concerns on medical social media by hashtag^a^: #IRad vs #CardioTwitter.”

		#IRad, n (%)	#CardioTwitter, n (%)	Fisher exact *P* value	Chi-squared with Yates correction *P* value
**#IRad vs #CardioTwitter**					
	Patient privacy	7 (1.4)	6 (1.2)	≥.99	.78
	Patient dignity	3 (0.6)	1 (0.2)	.62	.62
	Information accuracy	3 (0.6)	0 (0)	.37	.62
	Conflict of interest	18 (3.6)^b^	4 (0.8)^b^	.004^b^	.005^b^
	Justice and equity	0 (0)	0 (0)	≥.99	≥.99
	Interprofessional respect	8 (1.6)^b^	1 (0.2)^b^	.04^b^	.04^b^

^a^Comparisons reflect the composite of major and minor concerns for each scale criterion. *P*<.05 on a 2-tailed analysis was considered significant.

^b^Comparisons that are significant.

## Discussion

### Principal Results

This study sought to develop a scale to characterize and quantitate ethical issues on SoMe and then apply the scale to 3 different SoMe communities based on Twitter hashtags. Although some guidelines and opinion pieces exist describing potential ethical issues on SoMe, to the best of the authors’ knowledge, no scales had been created, making it difficult to assess the prevalence of ethical issues and guide efforts to mitigate potential harm [10]. This is important not only because of legal implications, but this behavior can exacerbate existing hierarchies and damage mutual trust.

The scale proposed in this study was developed via a structured deductive and inductive approach. Key domains were identified based on literature review as well as qualitative interviews, consistent with best practices in scale development [15,16]. This helped ensure that the scale was comprehensive and perceived as valid. Interrater agreement and correlation were good but likely limited by the qualitative nature of these assessments. The lack of interrelation between domains is not unexpected. A post with a patient privacy concern would not necessarily be more likely to have a conflict of interest as well.

Application of the scale to Twitter posts with #MedTwitter, #CardioTwitter, and #IRad yielded a couple of important observations. First, the prevalence of ethical concerns is low, often around 1% (n=5) across domains. However, such a number is not insignificant. According to Symplur software, there are approximately 5000 to 8000 posts per day made using #MedTwitter, equating to approximately 50-80 ethically concerning posts per day. These findings are similar to a 2011 study of over 5000 general tweets from health care providers, which found 3% of tweets were unprofessional and 0.7% were concerning for breaches in patient privacy [17].

A second interesting observation was how the prevalence of ethical concerns varied across the 3 groups of posts analyzed. For example, posts with the specialty-specific hashtags #CardioTwitter and #IRad had more patient privacy and conflict of interest concerns than general #MedTwitter posts. This may be due to a higher likelihood of posting specific patient cases in specialty-specific communities to illustrate an approach or solicit recommendations compared to the general #MedTwitter community. Posts with conflict of interest were also most prevalent in #IRad posts, which may be due to IR being a more procedural specialty than cardiology in general, and a specialty whose professional identity is closely tied to specific procedures and devices rather than patient populations [18]. Previous authors have observed similar variations in posts across specialties. The dominating content among IR posts tends to be images of an intervention performed on a patient to share new techniques or gather recommendations for superior approaches [19]. In contrast, cardiology posts are dominated by short synopses of trending research papers with reactive commentary [20]. However, interventional cardiology posts can share similar traits to IR [20,21], likely accounting for some of the overlap in the ethical issues among these posts.

### Practical Implications

The persistence of posts with ethical issues among medical professionals and trainees invites evaluation of current social media training programs. The domains in the scale offer a useful framework with validated language and examples to offer caution against ethical concerns that go beyond HIPAA violations. The framework can also foster a mental model to assist in evaluating personal tweets before publishing a post. This is important as once a post is made; it is difficult to retract it completely before it is shared or copied by other users.

The results from this study also provide a foundation for evidence-based social media guidelines by professional bodies and specialty-specific societies. As demonstrated by differences in the prevalence of ethical concerns between #CardioTwitter and #IRad, not all ethical issues are equally problematic, and with this data, guidelines can be tailored to the target group. This scale can be applied to hashtags used by other specialists to uncover trends in ethical issues and address those weak points more specifically. For example, social media statements for interventional radiologists may include more specific and detailed guidance on avoiding conflict of interest concerns.

From an academic perspective, the scale and methodology described in this study offer a way to assess the efficacy of interventions aimed at reducing the frequency of ethical issues on SoMe. Previously, there were limited ways to quantify and characterize the landscape of SoMe professionalism. However, now it is possible to perform pre- and poststudies with a specific intervention of interest.

Although this study focused on the application of the professionalism scale to Twitter posts as a proof of concept, the principles could be translated to other platforms as they do not include any evaluation metric that is inherent to Twitter, since the development of the scale was independent of any specific platform. From a validation perspective, this translation would be easiest for platforms that mimic Twitter by using a combination of texts and images, such as Facebook and Instagram posts. Importantly, videos were not assessed in this study, which would be of interest in analyzing Reels, TikTok, and YouTube videos. However, the methodology of this study can be applied to these different social media contexts to assess the generalizability of the scale.

### Limitations and Future Directions

This study had important limitations. The scale provides a good estimate of the prevalence of ethical issues, but it is not a thorough investigation of whether a given issue definitively exists especially for domains like conflict of interest that are challenging to verify without collateral information. Although the scale development incorporated input from a diverse group of clinicians and trainees in terms of training level, specialty, and gender identity, the sample was a small convenience sample from academic settings that could have missed important input from other clinicians in different contexts, for example, private practice. The sample was limited to posts in English from North America due to language restrictions and greater cultural familiarity. However, this may limit the external validity of the scale and results in other cultures. The authors relied on self-described Twitter biographies to limit posts to health care professionals, which could have been inaccurate.

To address some of these limitations, future steps to continue improving the scale would include expanding the sample to include more physicians and trainees from private practice, community hospitals, and primary care so that these additional perspectives can further refine the scale. Additionally, although the Cohen κ for interrater reliability already suggests good agreement, there may be domains with greater discrepancies than others. The language of these domains can be made more precise or explicit based on a bigger sample feedback to potentially improve consistency. Lastly, a comparison among different platforms would help directly assess if scale validity transcends social media contexts.

### Conclusions

The developed SoMe ethics scale is reliable, relevant, and concisely captures the myriad ethical tensions that can arise on these platforms. Ethical issues are present in a small but meaningful percentage of posts among health care professionals, which vary in important ways across different specialties and professional groups. The authors hope this scale will allow researchers to better characterize and assess the prevalence of ethical issues on SoMe while guiding more targeted interventions to mitigate these issues.

## Abbreviations

HIPAA: Health Insurance Portability and Accountability Act
IR: Interventional Radiology
SoMe: medical social media
